# Prevalence of overweight/obesity and related factors in Keerqin District, Tongliao City: A cross-sectional study

**DOI:** 10.1371/journal.pone.0282414

**Published:** 2023-08-04

**Authors:** Huiying Zhuang, Limei Wang, Fengying Wang, Yu Wang, Geisi Tang, Honglin Zhao

**Affiliations:** 1 Inner Mongolia University for the Nationalities, Tongliao, Inner Mongolia, P.R. China; 2 Key Laboratory of Zoonose Prevention and Control at Universities of Inner Mongolia Autonomous Region, Hohhot, P.R. China; 3 CDC: Centers for Disease Control and Prevention, Tongliao, Inner Mongolia, P.R. China; Public Library of Science, UNITED KINGDOM

## Abstract

**Objective:**

This study aimed to analyze the prevalence of overweight/obesity and the factors influencing these conditions among 9- to 18-year-old adolescents in Keerqin District of Tongliao City. We explored whether overweight/obesity is accompanied by differences in eating habits, lifestyle, and mental health.

**Methods:**

A cross-sectional survey was administered to 1,736 adolescents in November 2020. A physical examination was performed for each participant, and an online questionnaire was adopted to collect information. The association of several risk factors with overweight/obesity was explored using a logistic regression model.

**Results:**

The prevalence of overweight/obesity in the study population was 43.32%. The risk of overweight/obesity was higher among nonresident students (odds ratio [OR] = 1.564, 95% CI = 1.182–2.069) who had an average of 3–4 (OR = 2.164, 95% CI = 1.087–4.308) or 5 or more (OR = 2.114, 95% CI = 1.376–3.248) PE classes per week. The risk of overweight/obesity was lower among girls (OR = 0.485, 95% CI = 0.396–0.593), students aged 15–16 years (OR = 0.288, 95% CI = 0.135–0.617) and those aged 17–18 years (OR = 0.282, 95% CI = 0.124–0.639), students who ate sweets more than once a week (OR = 0.570, 95% CI = 0.366–0.887), students who spent less than 1 hour per day on the computer each week (OR = 0.776, 95% CI = 0.620–0.971), students with depressive symptoms (Center for Epidemiologic Studies Depression Scale [CES-D] score ≥ 16) (Model 2: OR = 0.618, 95% CI = 0.385–0.990; Model 3: OR = 0.623, 95% CI = 0.388–1.000), and students with depressed affect (Model 2: OR = 0.921, 95% CI = 0.877–0.967; Model 3: OR = 0.929, 95% CI = 0.885–0.976).

**Conclusion:**

Overweight/obesity was influenced by eating habits and lifestyle factors. In addition, overweight/obesity adolescents had a lower risk of depressed than those with normal weight.

## Introduction

The global prevalence of adolescent obesity has significantly increased in the past few decades. The prevalence of overweight and obesity among children and adolescents aged 5–19 years increased from 4% in 1975 to more than 18% in 2016 [1]. Overweight and obesity are the fifth leading cause of death worldwide, accounting for approximately 3.4 million deaths each year [2]. The incidence of overweight and obesity among children significantly increased in developing countries [3, 4]. Approximately 35 million children presented with overweight or obesity in 2014. The prevalence rates of overweight and obesity in Chinese children were 12.2% and 7.3%, respectively, in 2014 [3]. A study by Yuju C et al. showed that the prevalence rates of overweight and obesity in Chinese children were 14.4% and 11.9% in 2016 [5]. Obesity is associated with an increased risk of a variety of chronic diseases, including diabetes, cardiovascular disease (CVD), hypercholesterolemia, asthma and cancer [6]. Timely control of overweight can reduce the risk of obesity and obesity-related diseases in adults in the future [7]. Therefore, studies should identify the risk factors for overweight and obesity among adolescents to address this serious public health problem.

Epidemiological evidence shows that high energy intake, low intake of fruits and vegetables, and low physical activity are risk factors for overweight and obesity [8–10]. Diet is a key factor in the development of obesity [11]. The living standards in China have greatly improved owing to the rapid development of the economy, substantially changing people’s way of life. High-calorie and high-fat foods have become more common in children’s diets [12]. The presence of numerous high-energy foods in the modern diet is associated with an increased risk of lifestyle diseases [13]. These factors are the main causes of childhood obesity [14]. In addition to changes in dietary patterns, people are less reliant on cycling and walking, and the level of physical activity has declined recently [15]. The overall level of physical activity of the young population worldwide is very low, and the absence of physical activity is a major public health concern in both developed and developing countries. Studies have reported that the overall level of physical activity of children and adolescents in most countries is generally low [16, 17]. However, the level of sedentary behavior is high [18, 19], and the prevalence of obesity has been increasing recently [3, 20]. A recent study revealed that lack of sleep is associated with a high risk of obesity [21, 22]. In a cross-sectional study including 66,817 subjects [23], Wu et al. found that sleep duration of 7–8 hours had the lowest impact on the risks of overweight and obesity. Notably, sleep durations below or above the optimal range increased the risks of overweight and obesity among the participants. The quantity and quality of sleep are affected by the use of electronic devices before going to bed [24].

Most studies investigating the relationships among mental health, harmful behavior, and obesity have focused on children and adolescents. Clinical psychological studies have shown that obese children and adolescents often have psychological symptoms such as impaired self-consciousness, low self-esteem, lower happiness and life satisfaction [25], increased feelings of inferiority and anxiety [26], and increased depression [27]. Youth with overweight and obesity are more likely to experience harmful behavior, including relational, verbal, and physical victimization, than their normal-weight counterparts [28].

A study on adolescents who attended a weight-loss camp reported that 71% of participants developed injuries at school in the past year because of their weight, and more than 1/3 of subjects reported that such injuries had occurred for more than five years [29]. Another study conducted in Japan reported that 23.9% of adolescents were made fun of because of their body size and that students who thought they were obese were more likely to be ridiculed [30]. These studies showed that adolescents are vulnerable to weight discrimination in different environments. This may affect the health of obese children; thus, there is a need to explore strategies for early implementation of interventions.

Adolescence is a period characterized by drastic changes. Physical and psychological growth significantly affect individual lifestyle and health behavior and ultimately alter health in adulthood [31]. In some studies, depressed adolescents were at increased risk for the development and persistence of obesity in the USA [32], but among Korean [33] and island-dwelling Puerto Rican [34] adolescents, overweight and obesity are not related to high levels of depressive symptoms.

Only a few studies have explored the relationship of mental health and lifestyle in Chinese adolescents aged 9–18 years with risks of overweight and obesity. Adolescence is a critical period of growth and development. It is important to explore the associations among eating habits, lifestyle, psychological factors and body mass index (BMI) for the development of obesity prevention strategies. The present study showed an association between overweight/obesity and psychological factors that suggests the need to better evaluate depression. This study aimed to explore the risk factors for overweight and obesity among individuals aged 9–18 years in Keerqin District, Tongliao City, China. The present study proposes effective methods for reducing the incidence rates of overweight and obesity in adolescents.

## Materials and methods

### Study area

Tongliao is located in eastern Inner Mongolia, China and covers 58,863 km^2^. It contains eight counties with a population of 3.17 million in 2019. The topography is high in the south and north and low in the middle, with an altitude range of 120–1,400 m. Tongliao is an important pastoral area in China. The people in Tongliao mainly consume beef, mutton and dairy products.

### Study design and inclusion criteria

The present study aimed to provide recommendations for the requirements regarding school health-related work as outlined in the Healthy China 2030 Plan. The study began in May 2020 and ended in December 2020. The survey was distributed through stratified random cluster sampling from November to December 2020. Stratified sampling was adopted to select 2 primary schools, 2 middle schools and 2 high schools. Subsequently, stratified cluster sampling was adopted to randomly select 2 classes in each grade at each school. For this study, a random sample of 6 schools, including primary school students (grades 4–6), middle school students (grades 7–9), and high school students (grades 10–12), was selected. The inclusion criteria were as follows: students able to complete various examinations and questionnaires. The exclusion criteria were as follows: 1) diagnosed with heart disease, hypertension, diabetes, anemia, allergic asthma or other chronic diseases; 2) unable to complete the physical examination and questionnaire on time; 3) verbally declined to participate in the survey; and 4) aged < 9 years and > 18 years. A total of 1,757 primary, middle, and high school students were recruited for the study. Students who did not meet the criteria were excluded, resulting in a final sample size of 1,736 primary, middle, and high school students.

### Data acquisition

#### Anthropometric measurement

Weight and height were determined for all participants at school. These parameters were determined with shoes off, and the subjects wore school uniforms. Weight and height measurements were taken twice with a measurement device (EF06B, HC, Shanghai, China), and the average of the 2 values was adopted. Standardized equipment was used to measure height (accurate to 0.1 cm) and weight (accurate to 0.1 kg). Body mass index (BMI) was calculated as weight (kg)/height (m)^2^. The World Health Organization (WHO) guidelines were adopted to calculate BMI and BMI z scores for age- and sex-specific reference ranges for children aged 5–19 years. Normal weight was defined as within 1 SD of the mean, and overweight/obesity was defined as > 1 SD from the mean [35].

#### Demographic information

Data including sex, age (five age groups: 9–10, 11–12, 13–14, 15–16, and 17–18), school (primary school, middle school, and high school) and residence were obtained for all participants.

#### Lifestyle data

Data on the lifestyle of participants included dietary intake in the past 7 days, including intake of sugary drinks (including coke, iced black tea, orange juice, and nutrition express), sweets (including candy, cake, chocolate, and sweet soup), fried food (including fried dough, French fries, and fried chicken wings), fresh fruit (excluding canned fruit), vegetables (both raw and cooked), and breakfast. International guidelines for physical activity for children and adolescents (aged 5–17 years) [36] recommend daily physical activity, including mild and moderate to intense physical activity and medium- or high-intensity aerobic physical activity. Physical activity data included frequency of medium-to-high-intensity exercise (leading to rapid heart rate and loss of breath, such as running, basketball, football, and swimming) in the past 7 days, weekly PE class frequency, and daytime outdoor activities. Electronic screen usage data included time spent on televisions and computers in the past 7 days. Sleep duration was determined according to the recommendation of the National Sleep Foundation [37]. At least nine hours of sleep a day is considered adequate, and less than nine hours a day is considered insufficient for people aged 9 to 18 years.

#### Psychological factors

Psychological factors were evaluated based on the Center for Epidemiologic Studies Depression Scale (CES-D) [38], which comprises a total of 20 items. In the present study, depressed affect (8 questions), positive affect (4 questions), somatic symptoms (6 questions) and interpersonal relationships (2 questions) were evaluated. Answers are provided on a 4-point scale as follows: none or occasionally = 0 points, sometimes = 1 point, frequently = 2 points, mostly or always = 3 points. The range of scores is 0–60 points. A high score indicates severe depression, whereas a score of 16 represents the cutoff point for the identification of major depressive disorder [39]. Positive affect items were reverse scored as follows: no or occasionally = 3 points, sometimes = 2 points, frequently = 1 point, and mostly or always = 0 points. Factor analysis has revealed four subdimensions of depressive symptoms (depressed affect, lack of positive affect, somatic symptoms and interpersonal problems) [39]. Therefore, depression was assessed in three ways as follows: according to the depressive cutoff point (CES-D score ≥ 16), the total CES-D score, and the four subdimensions of the CES-D. Evaluation of psychological factors was performed only for middle school and high school students.

### Quality control

All the researchers in this study performed the study in May 2020. The investigators were health professionals. They received training on the purpose, methods and processes of the investigation from May to October 2020 and mastered the testing methods. They conducted the survey only after passing the training examination. A pilot test was performed, and the wording, logic and item order of the questionnaire were improved before the survey.

### Statistical analysis

All data were entered into a spreadsheet by the school health professionals. After alignment correction, the data were analyzed using SPSS 23.0 software. Concentration and discretization trends of quantitative variables were expressed as the mean ± standard deviation. Categorical variables were compared using Pearson’s χ^2^ test. Independent-samples t tests and Mann‒Whitney U tests were adopted to compare the total score of the CES-D and the four subdimension scores between normal-weight and overweight/obesity groups.

Binary logistic regression was adopted to explore the influencing factors of overweight/obesity. Single factor analysis was conducted to evaluate the potential correlation between overweight/obesity and the studied variables. A binary logistic regression model was adopted to evaluate all statistically significant factors obtained from the univariate analysis. Dependent variables were grouped according to weight (normal weight and overweight/obesity). Model 1 did not control for any confounding factors in the analysis of depression severity. Model 2 adjusted for adolescents’ personal characteristics, including gender (reference group: male) and age (in years). Model 3 additionally adjusted for two confounding factors, grade (reference group: middle school) and resident status (reference group: nonresident), and the method of backward stepwise selection was used for logistic regression analysis. The odds ratio (OR) and the corresponding 95% confidence interval (CI) were calculated to identify the risk factors for overweight/obesity. In the model, OR > 1.0 and P < 0.05 indicated that the parameter was a risk factor for overweight/obesity. In contrast, OR < 1.0 and P < 0.05 indicated that the parameter was a protective factor against overweight/obesity. P < 0.05 indicated statistically significant differences.

## Results

A total of 1,736 participants aged 9 to 18 years were included in the present study. The prevalence rate of overweight/obesity in males was higher than that in females (P < 0.001, Table 1). The highest prevalence rate of overweight/obesity was observed in 9-10-year-olds (58.57%). The lowest prevalence rate of overweight/obesity was observed in 15-16-year-olds (27.96%). The findings showed significant differences in body weight among age groups (P < 0.001). The prevalence rates of overweight/obesity according to school were 58.39% (primary school), 41.36% (middle school) and 29.08% (high school). Analysis showed significant differences in body weight among the school groups (P < 0.001). The prevalence rate of overweight/obesity in students who did not live at school was higher than that in students living at school (P < 0.001).

**Table 1:** Demographic characteristics and weight status of primary, middle, and high school students.

Variable		Normal weight (n/%)	Overweight/obesity (n/%)	P
Participants	Total	984(56.68)	752(43.32)	
Gender	Male	434(48.65)	458(51.35)	**<0.001**
	Female	550(65.17)	294(34.83)	
Age	9–10	133(41.43)	188(58.57)	**<0.001**
	11–12	196(44.95)	240(55.05)	
	13–14	260(60.89)	167(39.11)	
	15–16	237(72.04)	92(27.96)	
	17–18	158(70.85)	65(29.15)	
Grade	Primary school	243(41.61)	341(58.39)	**<0.001**
	Middle school	363(58.64)	256(41.36)	
	High school	378(70.92)	155(29.08)	
Live on campus or not	Yes	549(68.03)	258(31.97)	**<0.001**
	No	435(46.82)	494(53.18)	

**Fig 1:**
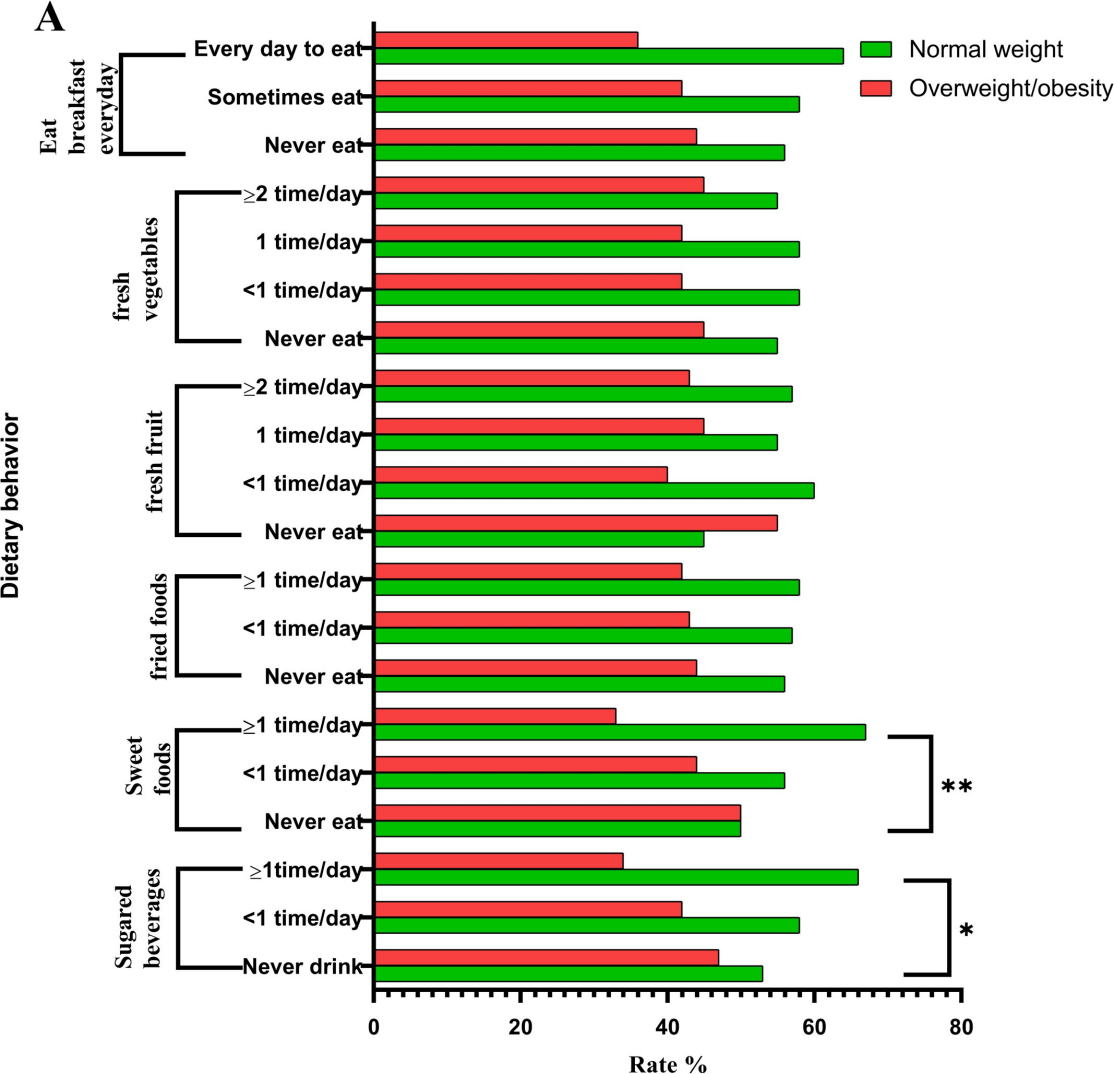
Comparison of dietary behavior and body weight among primary, middle, and high school students (A). *p<0.05; **p<0.01.

The comparison of dietary behaviors among primary, middle, and high school students is presented in Fig 1. The prevalence of overweight/obesity among primary, middle, and high school students decreased with an increase in the number of sugary drinks consumed the week before the study (P < 0.05). Furthermore, the prevalence rate of overweight/obesity was highest among students who had not consumed sugary drinks before the study (47.12%). In addition, the prevalence rates of overweight/obesity among primary, middle, and high school students significantly differed according to sweet consumption (never eating sweets, eating sweets < 1 time/day, and eating sweets ≥ 1 time/day in the week before the study) (P < 0.01). The prevalence rate of overweight/obesity was highest among students who had not eaten sweets the week before the study (49.57%). The findings showed no significant association of intakes of fried foods, fresh fruits, and vegetables or consumption of breakfast every day with body weight.

The results showed a significant association between the number of days of medium-and high-intensity exercise (P = 0.014, Table 2), the number of PE classes (P < 0.001), the time spent participating in outdoor activities during the day (P = 0.033), the time spent on computers every day (P = 0.009), and the average time spent sleeping each day (P < 0.001).

**Table 2:** Comparison of lifestyle factors and body weight among primary, middle, and high school students.

Variable		Normal weight (n/%)	Overweight/obesity (n/%)	P
How many days a week can you do at least 60 minutes of medium-and high-intensity exercise every day.	0 day	188(57.32)	140(42.68)	**0.014**
	1–2 days	270(52.94)	240(47.06)	
	3–4 days	192(53.19)	169(46.81)	
	5–6 days	174(60.21)	115(39.79)	
	7 days	160(64.52)	88(35.48)	
Average number of physical education classes per week	0 class	90(68.18)	42(31.82)	**<0.001**
	1–2 classes	667(59.50)	454(40.50)	
	3–4 classes	204(47.00)	230(53.00)	
	> 5 classes	23(46.94)	26(53.06)	
Outdoor activities on weekends within a week	< 1 hour	257(51.61)	241(48.39)	**0.033**
	1–2 (excluding 2) hours	338(56.52)	260(43.48)	
	2–3 (excluding 3) hours	155(62.25)	94(37.75)	
	≥ 3 hours	159(61.15)	101(38.85)	
	I don’t know	75(57.25)	56(42.75)	
How much time do you watch TV on average every day in a week?	I haven’t watch TV	231(58.04)	167(41.96)	0.266
	< 1 hour	382(53.65)	330(46.35)	
	1–2 (excluding 2) hours	191(58.05)	138(41.95)	
	2–3 (excluding 3) hours	87(59.59)	59(40.41)	
	3–4 (excluding 4) hours	27(55.10)	22(44.90)	
	≥ 4 hours	66(64.71)	36(35.29)	
How long do you watch the computer every day in a week?	I haven’t watched the computer.	460(53.12)	406(46.88)	**0.009**
	< 1 hour	322(60.41)	211(39.59)	
	1–2 (excluding 2) hours	113(61.41)	71(38.59)	
	2–3 (excluding 3) hours	43(55.84)	34(44.16)	
	3–4 (excluding 4) hours	12(42.84)	16(57.14)	
	≥ 4 hours	34(70.83)	14(29.17)	
Average daily sleep time	< 7 hours	245(65.68)	128(34.32)	**<0.001**
	7.0–8.9 hours	570(56.10)	446(43.90)	
	9–11 hours	161(48.35)	172(51.65)	
	> 11 hours	8(57.14)	6(42.86)	

Analysis of demographic characteristics showed that the risk of overweight/obesity in girls was 0.485 times lower than that in boys (95% CI = 0.396–0.593, P < 0.001, Table 3). Furthermore, the risk of overweight/obesity of students aged 15–16 years was 0.288 times lower (95% CI = 0.135–0.617, p = 0.001) and that of students aged 17–18 years was 0.282 times lower (95% CI = 0.124–0.639, p = 0.002) than that of students aged 9–10 years. The risk of overweight/obesity of nonresident students was 1.564 times higher than that of resident students (95% CI = 1.182–2.069, p = 0.002). Analysis of dietary behavior showed that students who ate sweets more than once a week had a 0.570 times lower risk of overweight/obesity than students who never ate sweets (95% CI = 0.366–0.887, p = 0.013). The number of sugary drinks consumed was not significantly associated with overweight/obesity. Lifestyle analysis showed that the risk of overweight/obesity of students taking an average of 3–4 PE classes per week was 2.164 times higher (95% CI = 1.087–4.308, p = 0.028) and that of students taking 5 or more PE classes per week was 2.114 times higher (95% CI = 1.376–3.248, p = 0.001) than that of students taking 0 PE classes. The risk of overweight/obesity of students who spent less than 1 hour per day on the computer in a week was 0.776 times lower than that of students who did not use the computer (95% CI = 0.620–0.971, p = 0.027). These findings indicate that boys, 9- to 10-year-old students, students not living at school, students who never ate sweets, students attending an average of 3–4 PE classes per week or 5 or more PE classes and students who had not used a computer within the past week were at higher risk of overweight/obesity.

**Table 3:** Binary logistic regression analysis of factors related to overweight/obesity.

Variable		P	OR(95%CI)
**Demographic characteristics**			
Gender	Male		Referent
	Female	**<0.001**	0.485(0.396–0.593)
Age	9–10		Referent
	11–12	0.938	0.987(0.705–1.381)
	13–14	0.058	0.623(0.382–1.016)
	15–16	**0.001**	0.288(0.135–0.617)
	17–18	**0.002**	0.282(0.124–0.639)
Grade	Primary school		Referent
	Middle school	0.354	0.693(0.319–1.505)
	High school	0.128	0.610(0.322–1.154)
Live on campus or not	Yes		Referent
	No	**0.002**	1.564(1.182–2.069)
**Dietary behavior**			
How many times have you had sugary drinks in a week.	Never drink		Referent
	< 1/day	0.274	1.352(0.787–2.322)
	≥ 1/day	0.485	1.201(0.719–2.006)
How many times have you had sweets in a week.	Never eat		Referent
	< 1/day	0.257	0.839(0.619–1.137)
	≥ 1/day	**0.013**	0.570(0.366–0.887)
**Lifestyle**			
How many days a week can you do at least 60 minutes of medium-and high-intensity exercise every day.	0 day		Referent
	1–2 days	0.159	1.230(0.922–1.641)
	3–4 days	0.368	1.153(0.845–1.574)
	5–6 days	0.830	0.964(0.689–1.348)
	7 days	0.168	0.782(0.551–1.109)
How many PE classes per week on average.	0 class		Referent
	1–2 classes	0.091	1.408(0.947–2.094)
	3–4 classes	**0.028**	2.164(1.087–4.308)
	≥ 5 classes	**0.001**	2.114(1.376–3.248)
Time for outdoor activities during the day within a week	< 1 hour		Referent
	1–2 (excluding 2) hours	0.333	1.218(0.817–1.814)
	2–3 (excluding 3) hours	0.881	1.031(0.695–1.528)
	≥ 3 hours	0.338	0.804(0.515–1.256)
	I don’t know	0.448	0.844(0.543–1.309)
How long do you watch the computer every day in a week?	I haven’t watched the computer.		Referent
	< 1 hour	**0.027**	0.776(0.620–0.971)
	1–2 (excluding 2) hours	0.261	0.824(0.588–1.155)
	2–3 (excluding 3) hours	0.757	1.080(0.665–1.753)
	3–4 (excluding 4) hours	0.134	1.810(0.833–3.930)
	≥ 4 hours	0.094	0.573(0.299–1.099)
Average daily sleep time	< 7 hours		Referent
	7.0–8.9 hours	0.400	0.623(0.207–1.875)
	9–11 hours	0.745	0.835(0.281–2.481)
	> 11 hours	0.890	1.081(0.358–3.265)

**Fig 2:**
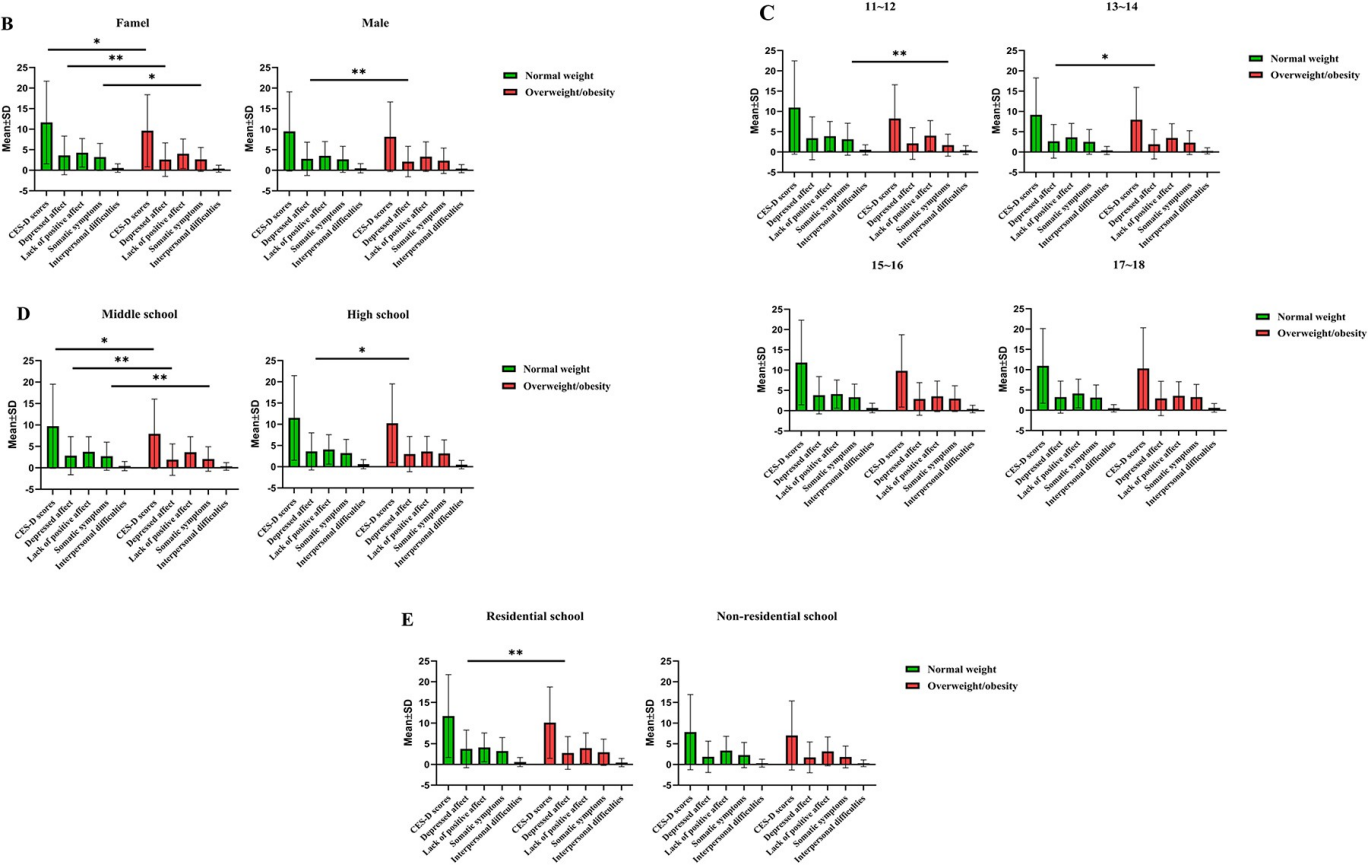
Difference in Center for Epidemiologic Studies Depression Scale (CES-D) scores according to (A) weight (normal weight and overweight/obese), (B) gender, (C) age, (D) school group, and (E) resident status. *p<0.05; **p<0.01.

The results showed that the CES-D total, depressed affect and somatic symptom scores in overweight/obese girls were lower than those in girls with normal weight (P < 0.05, Fig 2). The scores of depressed affect in overweight/obese boys were lower than those in boys with normal weight (P < 0.01). The scores of somatic symptoms in 11- to 12-year-old students were lower in overweight/obese students than normal-weight students (P < 0.01). Furthermore, the scores of depressed affect in 13- to 14-year-olds were lower in overweight/obese students than in normal-weight students (P < 0.05). The findings showed no significant difference in psychological factors between overweight/obese and normal-weight students aged 15–16 and 17–18 years. The CES-D total, depressed affect and somatic symptom scores in middle school students were lower in overweight/obese individuals than normal-weight individuals (P < 0.05). The scores of depressed affect in high school students were lower in overweight/obese individuals than normal-weight individuals (P < 0.05). The score of depressed affect in resident students was lower in overweight/obese individuals than normal-weight individuals (P < 0.05). Further analysis showed no significant difference in psychological factors between overweight/obese and normal-weight nonresident students.

Depressive symptoms (CES-D score ≥ 16) were significantly associated with overweight/obesity after adjusting for sex, age, school and living environment (confounding factors) (Model 2, OR = 0.618, 95% CI = 0.385–0.990, P *=* 0.045; Model 3, OR = 0.623, 95% CI = 0.388–1.000, P = 0.050; Table 4). Further analysis showed that depressed affect was significantly associated with overweight/obesity (Model 1: OR = 0.946, 95% CI = 0.917–0.977, P *=* 0.001). The association between depression and overweight/obesity was significant even after adjusting for sex, age, school and living environment (Model 2, OR = 0.921, 95% CI = 0.877–0.967, P = 0.001; Model 3 OR = 0.929, 95% CI = 0.885–0.976, P = 0.003). This indicates that depressed affect had the largest estimated effect on overweight/obesity of the four subdimensions of the CES-D.

**Table 4:** Binary logistic regression analysis of body weight and psychological factors.

Variable	Model 1		Model 2		Model 3	
	P	OR (95%CI)	P	OR (95%CI)	P	OR (95%CI)
Whether depressed			**0.045**	0.618(0.385–0.990)	**0.050**	0.623(0.388–1.000)
Depressed affect	**0.001**	0.946(0.917–0.977)	**0.001**	0.921(0.877–0.967)	**0.003**	0.929(0.885–0.976)

Model 1: no adjustment, Model 2: adjusted for age and gender, and Model 3: additionally adjusted for school and residential status.

## Discussion

The present results showed that the prevalence rate of overweight/obesity in Keerqin District of Tongliao City was 43.32%. This rate was significantly higher than that presented in the 2014 National Student Physique and Health Survey [40] (19.40%). Furthermore, it was higher than the rate obtained in surveys in Jiangxi Province (28.90%) [41] and Wuhan City (29.78%) [42] and was similar to that in Henan Province (42.30%) [43]. These findings indicate that studies should explore ways to manage overweight/obesity among adolescents in Keerqin District of Tongliao City.

Further analysis showed that the risk of overweight/obesity among 9- to 18-year-olds was higher in boys than in girls, which is consistent with the results obtained from population studies in other regions [44, 45]. Girls may pay more attention to their body image and consciously control their diet, thus reducing the incidence of obesity. On the other hand, boys are more likely to exercise, eat more, eat faster, prefer meat and high-energy foods and are less conscious of their body shape. The results showed that the prevalence of overweight/obesity decreased gradually with increasing age. The risk of overweight/obesity in adolescents aged 9–10 years was higher than that in adolescents aged 15–18 years. The incidence of overweight/obesity in adolescents aged 9–10 years was the highest compared with that in the other age groups. The incidence rates of overweight/obesity in primary school students were higher than those in middle and high school students, which is consistent with previous results [40, 46]. Younger adolescents have less knowledge about obesity, are more vulnerable to temptation of delicious food and have poor self-control [47]. In contrast, middle school students may be relatively more self-disciplined and can control their diet. In addition, middle school students are in the beginning of puberty and grow rapidly, especially in early adolescence, leading to a decrease in the incidence rates of overweight and obesity [48]. The findings indicate that 9- to 10-year-old primary school students is a key population for the prevention and control of overweight and obesity in children and adolescents. The results showed that adolescents who did not live on campus had a higher risk of being overweight/obese than those who lived on campus. This may be because resident students in China engage in daily morning exercises or because the management of diet for resident students is relatively strict. There are no reports of related studies in China; hence, further research is needed to define the relationship between obesity and resident status in students.

Poor eating habits are closely related to overweight/obesity in adolescents. The present findings indicated that sweets containing high-energy foods are protective factors against overweight/obesity, which is contrary to the results reported by Tambalis et al. [49] and Sobek et al. [50]. Adolescents who had not eaten sweets the week before the study had a higher risk of being overweight/obese than those who had eaten sweets once a day. This finding may be attributed to the decrease in meal intake due to an increase in sweet food intake. In addition, overweight/obese adolescents themselves may be aware that they are overweight and deliberately reduce their intake of sweets. One study reported that children and adolescents who do not consume snacks are more likely to be obese [51]. The relationship between sweet consumption and overweight/obesity should be explored further.

An appropriate amount of exercise effectively prevents the occurrences of overweight and obesity. The findings of the current study showed that the risk of overweight/obesity was not correlated with engaging in at least 60 minutes of medium- and high-intensity exercise and weekend outdoor activities. This needs to be further investigated. However, the prevalence rate of overweight/obesity was lowest in the group that engaged in at least 60 minutes of medium- and high-intensity exercise every day. The prevalence rate of overweight/obesity was highest in the group that engaged in at least 60 minutes of high-intensity exercise for 1–2 days a week. The prevalence rate of overweight/obesity was the lowest in students who participated in outdoor activities for 2–3 (excluding 3) hours a day and highest for students who participated in outdoor activities for less than 1 hour a day. Notably, the risk of overweight/obesity in students who took an average of 3–4 or 5 or more PE classes per week was higher than that of students who took 0 PE classes. This demonstrates that overweight/obese adolescents are more willing to take PE classes in school to achieve at least 60 minutes of medium- and high-intensity exercise and daytime outdoor activities, as recommended by the national physical education guidelines for primary schools and sedentary students [52]. Students are more likely to use PE facilities when they live at school. However, it is also possible that adolescents have a heavy academic burden, need to attend various tutoring classes on weekends, attend fewer outdoor activities, and have sufficient opportunities for physical activity at school. Therefore, overweight/obese students may have taken more PE classes to reduce their weight. Studies have reported that adolescents spend 3–4 (excluding 4) hours a day on the computer each week and that the incidence of overweight/obesity is highest among students who intensely use computers. In the present study, the risk of overweight/obesity in adolescents who spent less than 1 hour a day on the computer each week was lower than that of students who did not use the computer. Future studies should explore the relationship between the risk of obesity and using computers for long periods. Carson et al. [53] reviewed several cross-sectional studies and concluded that there was a correlation between obesity and screen time. Their results showed that the use of screens for a long time was correlated with a high risk of being overweight/obese. In addition, the findings indicated that using computers for a shorter time had lower risks of becoming overweight/obese compared with not using a computer. Therefore, spending less time on computers meets the needs of adolescents to use the computer and reduces the risk of being overweight/obese. Children who do not use computers for a long period may also spend their time on other activities, such as increased energy intake.

The number of overweight/obese adolescents is increasing owing to the rapid growth of China’s economy. Overweight/obesity affects the psychology of adolescents, leading to depression and anxiety symptoms. To date, only a few articles have explored the relationship between overweight/obesity and psychological factors in adolescents. Zavala et al. [54] and Zhao et al. [55] reported that depression is a key risk factor for overweight or obesity among adolescents. However, some studies have reported that overweight and obesity are not associated with depression in adolescents [56–58]. The results of the present study showed that overweight/obese adolescents had a lower risk of depression than normal-weight students after adjusting for confounding variables. Univariate analysis of the four subdimensions of the CES-D showed that depressed affect and somatic symptom scores were significantly correlated with overweight/obesity, whereas lack of positive affect and impaired interpersonal relationships were not significantly associated with overweight/obesity. The findings showed a significant difference in CES-D scores between male and female students and among students of different grades. The CES-D scores of overweight/obese adolescent female and middle school students were lower than those of normal-weight adolescents. This may be attributed to lifestyle factors of the students, eating habits, the environment or genetic factors. These findings should be further verified through scientific research. Notably, overweight/obese adolescents had a lower risk of depressed affect than normal-weight students, which is not consistent with findings from previous research. These findings indicate that overweight/obese adolescents have a lower risk of depression than normal-weight people. Further research should be conducted to explain these phenomena. Inconsistency of the results can be attributed to differences in sociodemographic characteristics of the samples, assessments of depressed affect, and height and weight measurements. Moreover, the typical diet of people in Inner Mongolia mainly includes beef, mutton and dairy products [59]. The incidence of overweight/obesity in this region is higher than that in other cities in China [60]. Overweight/obese adolescents may not view themselves as overweight, thus decreasing the rate of depressed affect. Longitudinal studies should be conducted to systematically explore the short-term and long-term relationship between depression and weight changes in Chinese adolescents. In the present study, we found that dimensions of depression, especially depressed affect, were associated with a lower risk of depression in overweight/obese adolescents than in normal-weight adolescents.

## Ethical statement

### Ethics approval and consent to participate

This study was approved by the Institutional Review Board at Inner Mongolia University for the Nationalities, A20200502 and adhered to the tenets of the Declaration of Helsinki. Participant data were anonymized, and no identifiable personal information was collected. Before enrollment in the study, each participant was informed of and understood the purpose of our investigation. Signed informed consent forms were obtained from the children’s parents before the anthropometric measurement and questionnaires.

## Conclusion

The findings of the present study showed that in Keerqin District of Tongliao City, adolescents have a higher rate of overweight/obesity. Moreover, males, 9-year-olds, 10-year-olds, primary school students, and students who do not live at school had higher risks of overweight/obesity. Adolescents who are overweight/obese may prefer PE classes to engaging in at least 60 minutes of medium-to-high-intensity exercise and outdoor activities. Spending less than 1 hour a day on the computer was associated with a lower risk of overweight/obesity than not using the computer. The results showed that overweight/obese adolescents had a lower risk of depression than normal-weight adolescents. These results provide a basis for the development of obesity prevention and control measures. Population-specific strategies should be included in future national intervention plans.

## Supporting information

S1 Table Comparisons of the Center for Epidemiologic Studies Depression Scale (CES-D) scores between different weight status groups. (DOCX)

S1 Data (XLSX)

S2 Data (XLSX)
