# Supplementary material for: Prevalence of overweight/obesity and related factors in Keerqin District, Tongliao City: A cross-sectional study
Source: PLoS One. 2023 Aug 4;18(8):e0282414. doi: 10.1371/journal.pone.0282414 (PMC10403123; doi:10.1371/journal.pone.0282414)
Supplement: S1 Table — (DOCX) [file pone.0282414.s001.docx]

**S1 Table. Comparisons of the Center for Epidemiologic Studies Depression Scale (CES-D) scores between different weight status groups.**

| Female | Normal weight  (n=399) | Overweight/obesity  (n=181) | *P* |
| --- | --- | --- | --- |
| CES-D scores | 11.22 ± 9.61 | 9.61 ± 8.77 | **0.028** |
| Depressed affect | 3.47 ± 4.56 | 2.58 ± 4.08 | **0.003** |
| Lack of positive affect | 4.13 ± 3.41 | 4.00 ±3.59 | 0.362 |
| Somatic symptoms | 3.15 ± 3.20 | 2.64 ± 2.92 | **0.047** |
| Interpersonal difficulties | 0.46 ± 0.96 | 0.38 ± 0.85 | 0.066 |
| Male | Normal weight  (n=342) | Overweight/obesity  (n=230) | *P* |
| CES-D scores | 9.45 ± 9.63 | 8.16 ± 8.48 | 0.175 |
| Depressed affect | 2.78 ± 4.04 | 2.12 ± 3.71 | **0.009** |
| Lack of positive affect | 3.51 ± 3.47 | 3.08 ± 3.59 | 0.332 |
| Somatic symptoms | 2.67 ± 3.16 | 2.33 ± 3.08 | 0.091 |
| Interpersonal difficulties | 0.48 ± 1.12 | 0.40 ± 0.98 | 0.286 |
| 11～12 years old | Normal weight  (n=89) | Overweight/obesity  (n=87) | *P* |
| CES-D scores | 10.95 ± 11.49 | 8.24 ± 8.32 | 0.155 |
| Depressed affect | 3.38 ± 5.28 | 2.10 ± 3.92 | 0.072 |
| Lack of positive affect | 3.89 ± 3.63 | 4.01 ±3.73 | 0.839 |
| Somatic symptoms | 3.14 ± 3.93 | 1.67 ± 2.71 | **0.005** |
| Interpersonal difficulties | 0.53 ± 1.24 | 0.45 ± 1.08 | 0.573 |
| 13～14 years old | Normal weight  (n=257) | Overweight/obesity  (n=167) | *P* |
| CES-D scores | 9.16 ± 9.11 | 7.95 ± 7.97 | 0.265 |
| Depressed affect | 2.64 ± 4.15 | 1.90 ± 3.61 | **0.043** |
| Lack of positive affect | 3.62 ± 3.40 | 3.46 ± 3.52 | 0.491 |
| Somatic symptoms | 2.50 ± 3.03 | 2.31 ± 2.94 | 0.462 |
| Interpersonal difficulties | 0.39 ± 1.01 | 0.27 ± 0.76 | 0.444 |
| 15～16 years old | Normal weight  (n=237) | Overweight/obesity  (n=92) | *P* |
| CES-D scores | 11.86 ± 10.44 | 9.80 ± 8.91 | 0.134 |
| Depressed affect | 3.81 ± 4.59 | 2.89 ± 4.01 | 0.055 |
| Lack of positive affect | 4.09 ± 3.84 | 3.54 ±3.76 | 0.133 |
| Somatic symptoms | 3.30 ± 3.26 | 2.95 ± 3.17 | 0.306 |
| Interpersonal difficulties | 0.66 ± 1.17 | 0.41 ± 0.91 | 0.054 |
| 17～18 years old | Normal weight  (n=158) | Overweight/obesity  (n=65) | *P* |
| CES-D scores | 10.95 ± 9.19 | 10.31 ± 10.02 | 0.500 |
| Depressed affect | 3.24 ± 3.95 | 2.91 ± 4.23 | 0.240 |
| Lack of positive affect | 4.13 ± 3.54 | 3.57 ± 3.47 | 0.264 |
| Somatic symptoms | 3.10 ± 3.13 | 3.23 ± 3.13 | 0.805 |
| Interpersonal difficulties | 0.48 ± 0.90 | 0.60 ± 1.07 | 0.767 |
| Junior middle school | Normal weight  (n=363) | Overweight/obesity  (n=256) | *P* |
| CES-D scores | 9.70 ± 9.80 | 7.93 ± 8.11 | **0.043** |
| Depressed affect | 2.84 ± 4.45 | 1.92 ± 3.66 | **0.004** |
| Lack of positive affect | 3.74 ± 3.51 | 3.62 ±3.64 | 0.502 |
| Somatic symptoms | 2.70 ± 3.27 | 2.06 ± 2.85 | **0.008** |
| Interpersonal difficulties | 0.42 ± 1.07 | 0.33 ± 0.89 | 0.484 |
| High school | Normal weight  (n=378) | Overweight/obesity  (n=155) | *P* |
| CES-D scores | 11.51 ± 9.96 | 10.23 ± 9.27 | 0.206 |
| Depressed affect | 3.61 ± 4.37 | 2.99 ± 4.15 | **0.045** |
| Lack of positive affect | 4.07 ± 3.46 | 3.60 ± 3.57 | 0.113 |
| Somatic symptoms | 3.22 ± 3.22 | 3.14 ± 3.17 | 0.763 |
| Interpersonal difficulties | 0.61 ± 1.08 | 0.49 ± 0.99 | 0.147 |
| Residential school | Normal weight  (n=534) | Overweight/obesity  (n=237) | *P* |
| CES-D scores | 11.71 ± 10.03 | 10.11 ± 8.62 | 0.091 |
| Depressed affect | 3.76 ± 4.54 | 2.77 ± 3.95 | **0.001** |
| Lack of positive affect | 4.12 ± 3.49 | 3.94 ± 3.67 | 0.377 |
| Somatic symptoms | 3.23 ± 3.29 | 2.94 ± 3.18 | 0.211 |
| Interpersonal difficulties | 0.59 ± 1.11 | 0.46 ± 1.00 | 0.066 |
| Non-residential school | Normal weight  (n=207) | Overweight/obesity  (n=174) | *P* |
| CES-D scores | 7.83 ± 9.08 | 7.01 ± 8.35 | 0.244 |
| Depressed affect | 1.85 ± 3.78 | 1.72 ± 3.72 | 0.277 |
| Lack of positive affect | 3.36 ± 3.44 | 3.17 ± 3.48 | 0.493 |
| Somatic symptoms | 2.28 ± 3.06 | 1.82 ± 2.64 | 0.087 |
| Interpersonal difficulties | 0.32 ± 0.97 | 0.30 ± 0.81 | 0.895 |

mean ± standard deviation
